# Prediction of central venous catheter-associated deep venous thrombosis in pediatric critical care settings

**DOI:** 10.1186/s12911-021-01700-w

**Published:** 2021-11-27

**Authors:** Haomin Li, Yang Lu, Xian Zeng, Cangcang Fu, Huilong Duan, Qiang Shu, Jihua Zhu

**Affiliations:** 1 Clinical Data Center, The Children’s Hospital, Zhejiang University School of Medicine and National Clinical Research Center for Child Health, 3333 Binsheng Road, Hangzhou, 310052 China; 2 Heart Center, The Children’s Hospital, Zhejiang University School of Medicine and National Clinical Research Center for Child Health, 3333 Binsheng Road, Hangzhou, 310052 China; 3 Department of Nursing, The Children’s Hospital, Zhejiang University School of Medicine and National Clinical Research Center for Child Health, 3333 Binsheng Road, Hangzhou, 310052 China; 4 The College of Biomedical Engineering and Instrument Science, Zhejiang University, Hangzhou, China

**Keywords:** Central venous catheter, Catheter-associated deep venous thrombosis, Machine learning, Prediction

## Abstract

**Background:**

An increase in the incidence of central venous catheter (CVC)-associated deep venous thrombosis (CADVT) has been reported in pediatric patients over the past decade. At the same time, current screening guidelines for venous thromboembolism risk have low sensitivity for CADVT in hospitalized children. This study utilized a multimodal deep learning model to predict CADVT before it occurs.

**Methods:**

Children who were admitted to intensive care units (ICUs) between December 2015 and December 2018 and with CVC placement at least 3 days were included. The variables analyzed included demographic characteristics, clinical conditions, laboratory test results, vital signs and medications. A multimodal deep learning (MMDL) model that can handle temporal data using long short-term memory (LSTM) and gated recurrent units (GRUs) was proposed for this prediction task. Four benchmark machine learning models, logistic regression (LR), random forest (RF), gradient boosting decision tree (GBDT) and a published cutting edge MMDL, were used to compare and evaluate the models with a fivefold cross-validation approach. Accuracy, recall, area under the ROC curve (AUC), and average precision (AP) were used to evaluate the discrimination of each model at three time points (24 h, 48 h and 72 h) before CADVT occurred. Brier score and Spiegelhalter’s z test were used measure the calibration of these prediction models.

**Results:**

A total of 1830 patients were included in this study, and approximately 15% developed CADVT. In the CADVT prediction task, the model proposed in this paper significantly outperforms both traditional machine learning models and existing multimodal deep learning models at all 3 time points. It achieved 77% accuracy and 90% recall at 24 h before CADVT was discovered. It can be used to accurately predict the occurrence of CADVT 72 h in advance with an accuracy of greater than 75%, a recall of more than 87%, and an AUC value of 0.82.

**Conclusion:**

In this study, a machine learning method was successfully established to predict CADVT in advance. These findings demonstrate that artificial intelligence (AI) could provide measures for thromboprophylaxis in a pediatric intensive care setting.

**Supplementary Information:**

The online version contains supplementary material available at 10.1186/s12911-021-01700-w.

## Background

Central venous catheters (CVCs) have revolutionized the care of patients requiring long-term venous access. The introduction of CVCs in pediatric intensive care units (ICUs) has been an important modality in the improved quality of care in critical patients [1]. Despite these advantages, more than 15% of patients receiving a CVC could develop complications [2], such as catheter malfunction, bloodstream infection, chylothorax, and CVC-associated deep venous thrombosis (CADVT) [3], which prolong the hospital stay and increase medical costs. CADVT constitutes 10% of all deep venous thromboses (DVTs) in adults and 50–80% of all DVTs among children [4], and nearly all DVT-related deaths in children are associated with CVCs [5]. In newborns, approximately 90% of venous thromboses are related to CVCs [6]. In pediatric patients, the presence of a CVC is the single most common risk factor for venous thromboembolism (VTE) [7, 8]. With the increasing use of CVCs, the incidence of CADVT has been on the rise. A significant increase in the rate by 30–70% has been reported among hospitalized children over the last 2 decades [9, 10]. Predicting CADVT events before they occur and taking necessary blood clot prevention measures in advance can help reverse this trend. However, current screening guidelines for venous thromboembolism risk, which are developed from incomplete pediatric data and extrapolated from adult data, have low sensitivity for CADVT in hospitalized children [11].

Machine learning is a form of artificial intelligence (AI) in which a model learns from examples rather than preprogrammed rules. Machine learning approaches can provide accurate predictions based on large, structured datasets extracted from electronic health records (EHRs) and have been applied in many clinical areas [12, 13]. Many machine learning methods have been rapidly developed to model complex and nonlinear effects and thereby improve prediction rules developed using standard statistical methods [14]. The advantage of completely data-driven learning without reliance on rule-based programming is that machine learning constitutes a reasonable approach. Therefore, this study applied machine learning methods to develop a model to accurately predict CADVT before it occurs.

## Methods

This retrospective study was approved by the Institutional Review Board of the Children’s Hospital of Zhejiang University School of Medicine, and the requirement for informed consent was waived. In this study, data were collected on pediatric patients who were admitted to the ICU of the 1900-bed Children’s Hospital, Zhejiang University School of Medicine, between December 2015 and December 2018. The clinical data from a total of 11,814 patients were recorded in a public pediatric intensive care (PIC) database [15]. Additional daily catheter assessment records and CVC-related adverse event reports were aligned with the PIC database. The inclusion criteria for this study were that patients received CVC placement for at least 3 days during the study period in one of the 4 ICUs of this children’s hospital. The exclusion criteria were incomplete patient-related data or patients with thrombosis either present before hospital admission or unrelated to CVC placement. The thrombus was confirmed with Doppler ultrasound at most time or computed tomography in very rare cases and recorded in the adverse event reports.

### Data collection and preprocessing

**Fig. 1:**
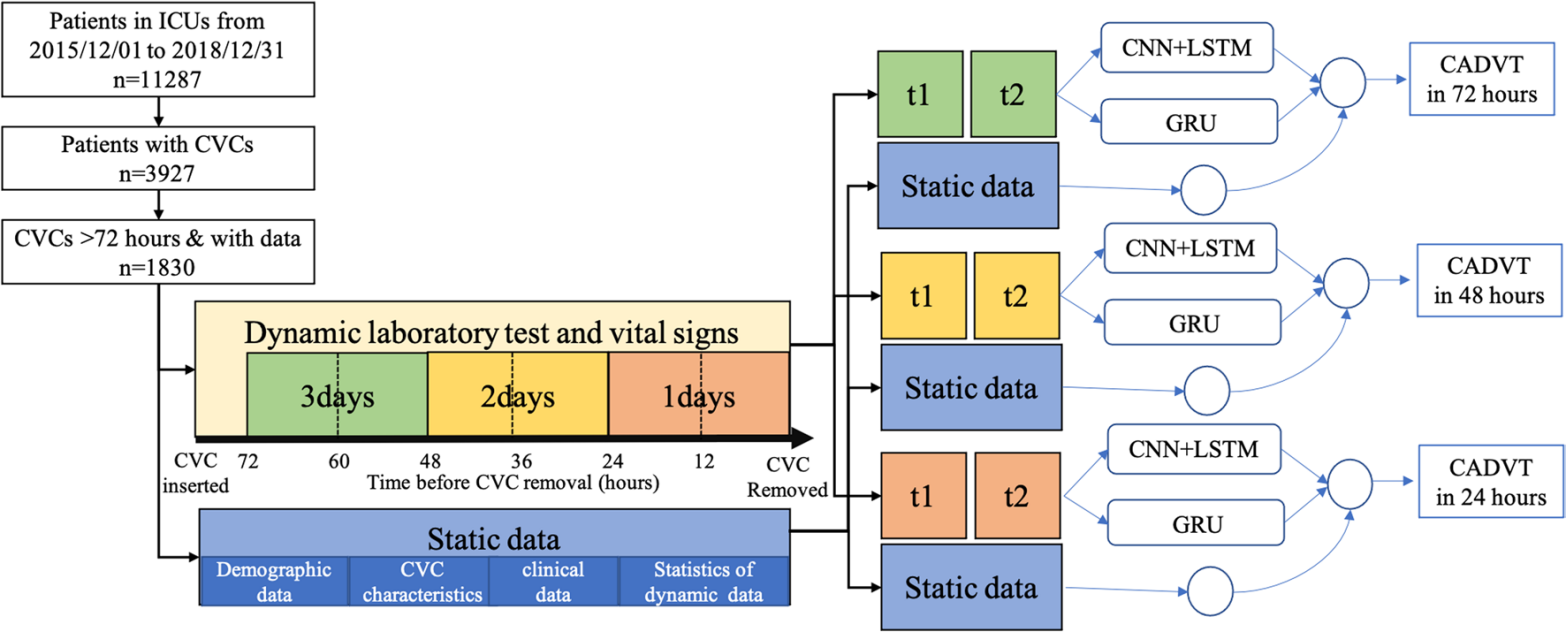
Flowchart for the study populations and methods

Patient-specific clinical data were collected from the PIC database, and CVC-related records were collected from different clinical information systems. The primary outcome of interest in the present study was the occurrence of CADVT. The variables included age, sex, primary diagnosis, surgery before CVC insertion, ICU, duration of CVC insertion, catheter-related characteristics (type and size) and administration of 6 types of drugs. Many dynamic temporal data, such as vital signs and laboratory test items, were also repeatedly collected, usually at different time intervals. For temporal data with multiple repeated measurements, statistical values such as the mean, standard deviation, minimum, median, and maximum are used as static feature and the original data were divided by 12-h windows to generate a time series. As a case–control design with three different lookback periods, only dynamic data collected 72 h before the CVC was removed were retained, and then the data were divided into three parts at 24-h intervals to test how far in advance a CADVT event can be predicted, as shown in Fig. 1. It is important to note that some time sensitive static data, such as the CVC dwell time and statistics generated from temporal data, differed at the 3 prediction time points. The details of these variables are shown in Additional file 1.

### Machine learning model

**Fig. 2:**
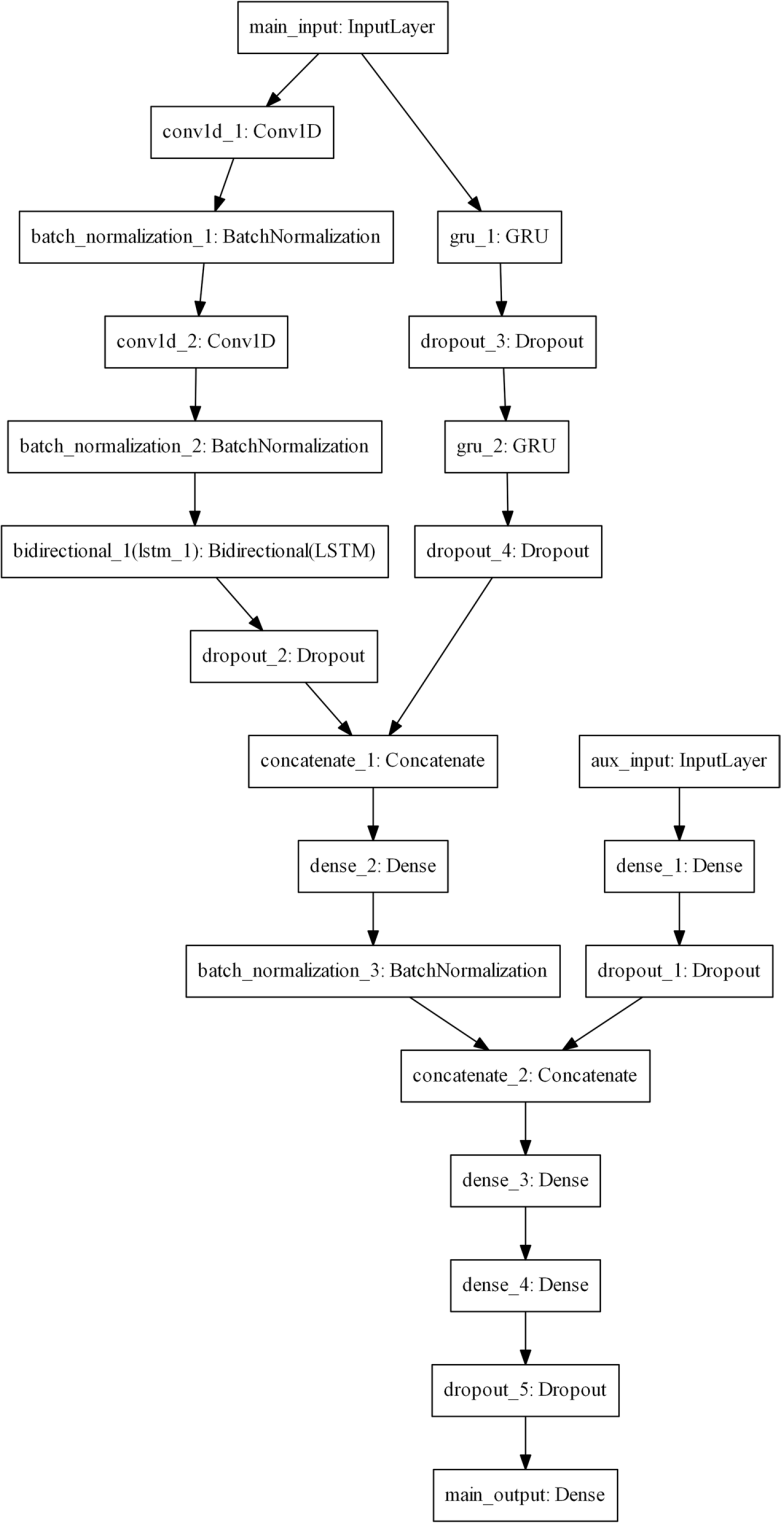
Structure of the proposed multimodal deep learning model

The traditional logistic regression (LR) model and two superior performance machine learning models, random forest (RF) and gradient boosting decision tree (GBDT), were used as three benchmark models that only use static data. Another benchmark model is a deep learning framework called the multimodal deep learning model (MMDL), which was recently proposed [16]. For simplicity, MMDL treats all the temporal dynamic features as one modality and all nontemporal static features as another modality using different deep learning models. In this study, we propose a novel multimodal deep learning model. The detailed architecture of the proposed model is shown in Fig. 2. Briefly, it accepts dynamic data using multiple recurrent neural network (RNN) models, including both long short-term memory (LSTM) and gated recurrent units (GRUs). The static features were fed into a standard feedforward neural network (FNN), and then the outputs of the FNN and dynamic feature model (LSTM + GRUs) were combined in a shared latent representation layer to predict CADVT. The positive patients (with CADVT) in the dataset accounted for approximately 15% of the total. The phenomenon of class imbalance or class skew can cause an unreasonable evaluation of the two-class classifier. The SMOTE method [17] was adopted as a processing method for unbalanced data before training. A fivefold cross-validation approach in which the dataset were randomly partitioned into 5 subsets of roughly equal size and each subset will be used as the testing set for a model trained on other 4 subsets in 5 rounds [18], was used in the evaluation. We used accuracy, recall, area under the ROC curve (AUC), and average precision (AP) to evaluate the discrimination of the prediction model. Brier score and Spiegelhalter’s z test were used measure the calibration of these prediction models [19]. All these experiments were conducted under the scikit-learn Python module.

### Statistical analysis

All statistical analyses were performed using packages in the Python and R programming environments. The patients were categorized according to whether they had experienced the primary outcome (i.e., CADVT). All differences in continuous data between patients with and without CADVT are reported as the mean ± standard deviation and were compared using the Mann–Whitney U test. All categorical data are reported as counts (percentages) and were compared using the chi-square test. For statistical hypothesis testing purposes, we considered a *p* value less than 0.05 to indicate significance.

## Results

Of 11,287 patients who were admitted to ICUs between December 2015 and December 2018, 3927 ICU admissions received CVC placement, but only 1830 children who met our inclusion criteria were included in this study. The detailed patient characteristics and CVC information are shown in Table 1. The mean age of the cohort was 24.3 ± 37.5 months, and 1046 (57.2%) patients were male. The spectrum of primary diagnoses included various cardiac, oncologic, infectious, gastrointestinal, and neurologic conditions. These were broadly categorized as congenital heart disease (CHD) [n = 595(32.5%)], infection or inflammation [n = 311(17.0%)], other congenital disease [n = 126(6.9%)], cancer [n = 121(6.6%)], nonmalignant pathology [n = 125(6.8%)], intracranial space-occupying lesion [n = 69(3.8%)], bleeding [n = 23(1.3%)], and other [n = 441(24.1%)] (detailed definitions of these disease groups are provided in Additional file 1: Table S2). Of these enrolled patients, 282 (15.4%) experienced CADVT, which was confirmed by duplex ultrasound or computed tomography. The average CVC dwell time was 147.22 ± 153.7 h.

**Table 1:** Patient characteristics stratified by CADVT status

Characteristic	Patients with CADVT n = 282 (15.4%)	Patients without CADVT n = 1548 (84.6%)	*P* value
Sex			< 0.001
Male	173 (61.3%)	873 (56.4%)	
Female	109 (38.7%)	675 (43.6%)	
Age (months)	41.1 ± 48.4	21.3 ± 34.3	< 0.001
Diagnosis			< 0.001
Bleeding	5(1.8%)	18 (1.2%)	
Cancer	14 (4.9%)	107 (6.9%)	
CHD	52 (18.4%)	543 (35.1%)	
Intracranial space-occupying lesion	22 (7.8%)	47 (3.0%)	
Nonmalignant pathology	6(2.1%)	119 (7.7%)	
Premature infant	0 (0.0%)	19 (1.2%)	
Infection/inflammation	75(26.6%)	236 (15.2%)	
Other congenital disease	8 (2.8%)	118 (7.6%)	
Other	100 (35.5%)	341 (22.0%)	
ICU admission			< 0.001
CICU	67 (23.8%)	703 (45.4%)	
NICU	4 (1.4%)	90 (5.8%)	
PICU	124 (44.0%)	232 (15.0%)	
SICU	87 (30.9%)	523 (33.8%)	
Catheter type			0.220
Single lumen	234 (80.4%)	1328 (75.1%)	
Double lumen	48 (19.6%)	220 (24.9%)	
Catheter model			< 0.001
18 G	58 (20.6%)	264 (17.1%)	
20 G	17 (6.0%)	33(2.1%)	
22 G	165 (58.5%)	1065 (68.8%)	
4.0 Fr	9 (3.2%)	33(2.1%)	
5.0 Fr	25 (8.9%)	134 (8.7%)	
Other	8 (2.8%)	19 (1.2%)	
CVC dwell time (h)	186.0 ± 254.4	140.2 ± 125.9	< 0.001
Surgery			< 0.001
True	174 (61.7%)	1261 (81.5%)	
False	108 (38.3%)	287 (18.5%)	

Patients with thrombosis were older, with a mean age of 41.1 ± 48.4 months versus 21.3 ± 34.3 months for those without thrombosis. CADVT occurred more frequent in boys than in girls. The odds of experiencing CADVT were significantly higher for patients with intracranial space-occupying lesions, bleeding, and infection/inflammatory diseases than for patients with other diseases. Many rare and risky diseases were classified as “other”, which accounted for more than one-third of CADVT events. The patient’s ICU type, history of surgery, catheter dwell time, and types and sizes of catheters were significantly associated with the occurrence of CADVT. Many vital signs and laboratory test items also showed significant differences between patients with and without CADVT (a detailed statistical analysis of all 56 temporal data points in all 3927 patients is available in Additional file 1: Table S1).

**Fig. 3:**
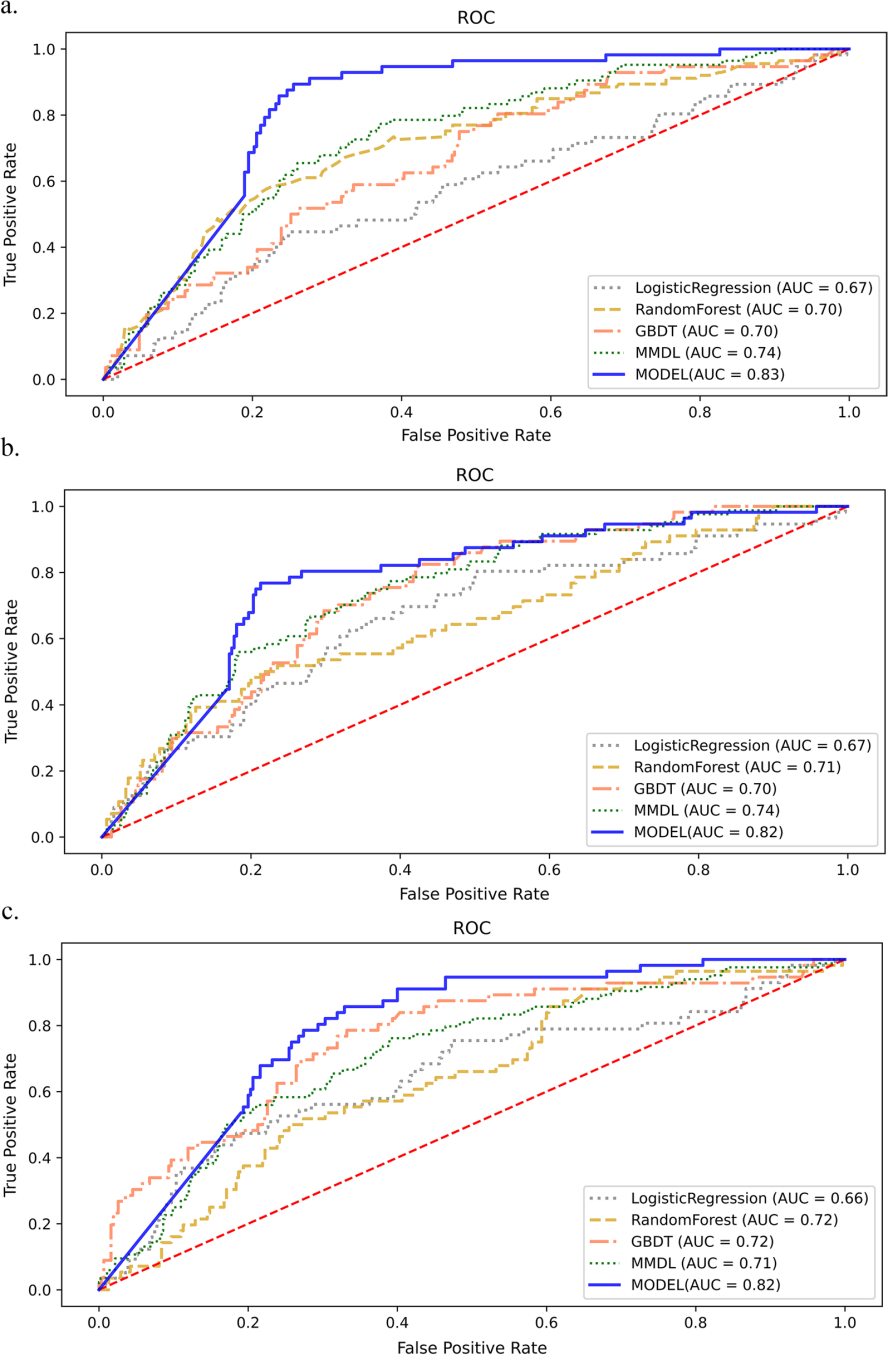
Comparison of AUCs among machine learning models at 3 time points. **a** Twenty-four hours before CADVT. **b** Forty-eight hours before CADVT. **c** Seventy-two hours before CADVT. The proposed model is shown as the solid blue line

The accuracy, recall, AUC and AP of the four benchmark models and the proposed model are shown in Table 2 and Fig. 3. The two machine learning models RF and GBDT achieved better performance than the traditional method LR at three time points, especially concerning recall. The MMDL, which uses additional temporal dynamic data, achieved better performance than the three benchmark models based on static data from the first two time points. The proposed model achieved the best prediction results in all 4 evaluation metrics at all 3 time points. The performance of each model did not differ significantly at the three time points. The proposed model can predict CADVT 72 h before it is discovered with an accuracy of 0.75 and a recall of 0.87.

**Table 2:** Performance of four benchmark models and the proposed model at three time points

Model	24 h in advance				48 h in advance				72 h in advance			
	Accuracy	Recall	AUC	AP	Accuracy	Recall	AUC	AP	Accuracy	Recall	AUC	AP
LR	0.64	0.61	0.67	0.27	0.66	0.62	0.67	0.28	0.65	0.57	0.66	0.29
RF	0.61	0.68	0.70	0.32	0.60	0.70	0.71	0.34	0.61	0.74	0.72	0.31
GBDT	0.57	0.73	0.70	0.31	0.57	0.75	0.70	0.29	0.60	0.75	0.72	0.32
MMDL	0.66	0.75	0.74	0.30	0.67	0.71	0.74	0.30	0.59	0.79	0.71	0.31
Model^a^	0.77	0.90	0.83	0.37	0.77	0.88	0.82	0.36	0.75	0.87	0.82	0.36

^a^The model proposed in this study

The calibration results of these models were shown in Table 3. The calibration and discrimination of these 5 models are not consistent. The RF model which achieved a smallest Brier score is the only one with Spiegelhalter’s test *p* value great than 0.05 in all three time points which means it is well calibrated. All other four models are not well calibrated. The LR and MMDL models will under-estimate the risk of CADVT. While, the GBDT and model proposed in this study will over-estimate it. If the model works as a risk calculator it will needs calibrate before implementation.

**Table 3:** Calibration results of models at three time points

Model	24 h in advance			48 h in advance			72 h in advance		
	Brier score	Spiegelhalter *z* score	Spiegelhalter *P* value	Brier score	Spiegelhalter *z* score	Spiegelhalter *P* value	Brier score	Spiegelhalter *z* score	Spiegelhalte *P* value
LR	0.149	18.39	0.00	0.147	18.31	0.00	0.145	17.96	0.00
RF	0.124	− 0.34	0.36	0.122	− 0.43	0.33	0.120	− 0.48	0.32
GBDT	0.141	− 5.57	2.53e^−8^	0.139	− 5.71	8.45e^−9^	0.138	− 5.76	1.7e^−8^
MMDL	0.188	2.53	1.61e^−2^	0.190	2.00	0.08	0.199	2.54	3.61e^−2^
Model^a^	0.167	− 4.94	1.70e^−6^	0.189	− 3.96	1.42e^−3^	0.177	− 4.55	1.41e^−3^

^a^The model proposed in this study

## Discussion

The prevalence of VTE has significantly increased across all age groups of hospitalized children, which has been attributed, in part, to the widely used CVCs in this population [10]. Given this critical and growing problem, several important pediatric organizations have developed initiatives to prevent VTE. However, a study showed that current screening guidelines for VTE risk in hospitalized children have low sensitivity (61%; 95% CI 51–70%) for identifying patients at increased risk of both CVC-associated and other VTE events [11]. It was also confirmed by the traditional LR model in this study that recall, which is the same as sensitivity, was approximately 61%. This means that approximately 40% of CADVT events will not be predicted. Traditional risk models are inadequate for this complex problem.

In this retrospective analysis, we evaluated the performance of different machine learning models to predict CADVT before it occurred at 3 time points. As a complicated task, the 3 machine learning models that only use static data did not achieve the desired predictive performance. A multimodal deep learning model called the MMDL, which can handle temporal data, exhibited an improved performance. Inspired by this finding, we believe that time-series dynamic data contain much more clinical information than static data or dynamic data-based statistics. For these reasons, we propose a new multimodal deep learning model that can provide deeper insight and learn shared latent representations for prediction tasks from both static and temporal dynamic data. The proposed model with an AUC > 0.82 can meet most needs of clinical applications. It allows clinicians to predict CADVT 3 days in advance. The ability to predict CADVT may allow patients to benefit from thromboprophylaxis or close surveillance [20]. The proposed deep learning models have the potential to be used as decision support tools for thromboprophylaxis. Although it is not too difficult to develop a machine learning model, data integration can be a challenge in practice for such a model that requires a large number of data features, especially dynamic data features. Prior to implementing predictive models in novel settings, analyses of calibration remain as important as discrimination, but they are not frequently discussed [21]. As many studies shown, a highly discriminative classifier (e.g., a classifier with a larger area under ROC curve) including widely used logistic regression model and several machine learning approaches such as Naïve Bayes, decision trees, and artificial neural networks all may not be well-calibrated [22, 23]. The calibration reported in this study also show many prediction models were not well calibrated.

The advantage of deep learning models is that they can receive a large number of data features at the same time and learn from them to obtain implicit correlations to serve complex prediction problems. In this study, the static data contained 143 features after one-hot encoding, and the dynamic data were in an n * 2 * 56 size 3D matrix. However, because the deep learning model is not highly interpretable, which factors and how they contribute to CADVT still need to be studied. Based on the contribution of different features, the model can be optimized and compressed to using fewer data input and with simpler network structure in practice. Furthermore, explainability is more important than accuracy, as it will identify which modified risk factor contributes to CADVT and what kind of measures could help to change the situation and determine a patient's individual risk. Explainable artificial intelligence (XAI), which is a set of processes and methods that allows human users to comprehend and trust the results and output created by machine learning algorithms, is also the focus of current AI research in medicine [24]. Some interpretation methods, such as SHAP [25], have been introduced to explain the output of machine learning models by computing each feature for the prediction. However, such an explainable framework works well on static features but not on latent temporal features.

Several studies have shown that different diseases have different risks of CADVT [26]. In this study, we found that patients with intracranial space occupying lesions have a particularly high risk of CADVT (OR 14.2 *p* value < 0.001 compared with CHD patients). Further analysis showed the dehydration agent, such as mannitol, glycerol fructose, and furosemide etc., that is widely used to reduce brain swelling and intracranial pressure, maybe contribute to the CADVT. For these reasons, intracranial occupying lesions are used independently as a feature. In addition, only the primary discharge diagnosis was used to label patient. It should include more diseases information in the future study. Therefore, for machine learning technology to be used to predict CADVT, patients in the training dataset should be collected from similar hospitals or the same hospital of the practice hospital due to the disease spectrum. Different hospitals with different populations with different diseases may require different models. Machine learning models trained on datasets from multiple centers should also consider hospitals as features. Furthermore, if the prediction risk probability were directly provided to clinician for clinical decision support, applying calibration model to estimates is needed.

This study had several limitations. First, as the temporal data were organized in fixed 12-h time windows, all the original temporal features of different clinical data were not retained. Furthermore, a more flexible learning model that could accept dynamic data at different time intervals should be developed. A scheme which uses forward well-defined index time from the catheter insertion was also suggested for future study. Second, as the proposed deep learning model was evaluated only in a single center with retrospective data in a case–control design, a larger evaluation using a cohort design is needed to demonstrate its broad applicability. The most important limitation of such a complicated multimodal deep learning model is its clinical explainability and its different calibration in different populations.

## Conclusions

In conclusion, children in ICUs are at high risk for CADVT, which occurs in approximately 15% of patients. In the CADVT prediction task, the model proposed in this paper significantly outperforms both traditional machine learning models and existing multimodal deep learning models at all 3 time points. The AI model was able to accurately predict the occurrence of CADVT 72 h before it was discovered with an accuracy of > 75%, a recall of > 87%, and an AUC of > 82%. This study demonstrates that AI could provide measures for thromboprophylaxis in pediatric intensive care settings.

## Supplementary Information

**Additional file 1.** Supplementary material.

## Data Availability

The datasets used and/or analyzed during the current study are available from the corresponding author on reasonable request.

## Abbreviations

CVC: Central venous catheter
CADVT: CVC-associated deep venous thrombosis
DVT: Deep venous thrombosis
VTE: Venous thromboembolism
AI: Artificial intelligence
PIC: Pediatric intensive care database
LR: Logistic regression
RF: Random forest
GBDT: Gradient boosting decision tree
MMDL: Multimodal deep learning model
RNN: Recurrent neural network
LSTM: Long short-term memory
GRU: Gated recurrent unit
FNN: Feedforward neural network
AUC: Area under the ROC curve
AP: Average precision
