# Supplementary material for: Prediction of central venous catheter-associated deep venous thrombosis in pediatric critical care settings
Source: BMC Med Inform Decis Mak. 2021 Nov 27;21:332. doi: 10.1186/s12911-021-01700-w (PMC8627017; doi:10.1186/s12911-021-01700-w)
Supplement: Supplementary file 1 — Additional file 1. Supplementary material. [file 12911_2021_1700_MOESM1_ESM.docx]

Prediction of Central Venous Catheter-Associated Deep Venous Thrombosis in Pediatric Critical Care Settings

Haomin Li ^a^, PhD, Yang Lu^a,b^, MS, Xian Zeng^a,b^, BS, Cangcang Fu^a^ ,MSN, Huilong Duan^b^, PhD, Qiang Shu^a^,MD, Jihua Zhu^a^, BN

**Affiliations:** ^a^The Children’s Hospital of Zhejiang University School of Medicine and National Clinical Research Center for Child Health, China; and ^b^The College of Biomedical Engineering and Instrument Science, Zhejiang University, China

Supplementary Material

Factors analyzed in this study

(1) The patient's age, gender, and whether or not the operation was performed;

(2) Central venous catheter type (single lumen / double lumen); catheter model (18G / 20G / 22G / 4.0Fr / 5.0Fr / other);

(3) Admission ICU (CICU / NICU / PICU / SICU);

(4) Disease diagnosis group（bleeding / cancer / CHD (congenital heart disease) / intracranial space-occupying lesion / nonmalignant pathology / premature infant / infection & inflammation / other congenital disease / other）.

(5) 7 vital signs (body temperature / pulse / heart rate / respiration / oxygen saturation / systolic blood pressure / diastolic blood pressure);

(6) 49 laboratory test items (Subject active partial thromboplastin time / plasma D-dimer / fibrinogen / international standardized ratio / normal control active partial thromboplastin time / normal control prothrombin time / normal control thrombin time / be Examiner's prothrombin time / subject's thrombin time / eosinophils / neutrophil absolute value / neutrophils / hematocrit / hemoglobin / lymphocyte absolute value / lymphocyte / average hemoglobin amount / average Hemoglobin concentration / average red blood cell volume / average platelet volume / platelet packed volume / platelet distribution width / platelet count / red blood cell count / red blood cell distribution width / white blood cell count / absolute value of basophils / absolute value of monocytes / monocytes / Eosinophil absolute value / calcium / chlorine / potassium / sodium / bicarbonate / methemoglobin / carbon dioxide partial pressure / pH / oxygen partial pressure / Standard base excess (SBE) / oxygen saturation / actual base residual / anion gap / carboxyhemoglobin / Glucose (electrode method) / Lactate / Thrombin time difference / Prothrombin time difference / Activated partial thromboplastin time difference).

(7) 6 drug therapy categories

(7.1) Anticoagulant drugs (heparin sodium injection / nadroparin calcium injection / low molecular weight heparin calcium injection / warfarin sodium tablets / aspirin enteric-coated tablets / dipyridamole tablets / clopidogrel bisulfate tablets);

(7.2) Procoagulant drugs (Hemocoagulase for injection / vitamin K1 injection / human coagulation factor Ⅷ / recombinant human coagulation factor Ⅷ for injection / recombinant human coagulation factor Ⅷa for injection / human prothrombin complex / human fiber Protein / Tranexamic acid injection / Tranexamic acid for injection / Tranexamic acid injection / Sulfenac injection / Poster pituitary injection);

(7.3) Dehydrating agent (20% mannitol injection / glycerol fructose sodium chloride injection / glycerol fructose injection / furosemide tablets / furosemide injection / 20% human albumin injection / concentrated sodium chloride Injection / 10% sodium chloride injection);

(7.4) Vasoconstrictive drugs (epinephrine hydrochloride injection / norepinephrine bitartrate injection / isoproterenol hydrochloride injection / phenylephrine hydrochloride injection);

(7.5) Vasodilators (dopamine hydrochloride injection / phentolamine mesylate for injection / phentolamine mesylate injection / nitroglycerin injection);

(7.6) Sedative drugs (diazepam injection / diazepam tablets / estazolam tablets / midazolam injection / clonazepam tablets / phenobarbital sodium for injection / phenobarbital tablets / Phenobarbital Sodium Injection).

**Table S1 Vital signs and lab tests stratified by patient CADVT status in all 3927 cases**

| **Item** | | **Patients with CADVT** | **Patients without CADVT** | **P-value** |
| --- | --- | --- | --- | --- |
| Vital sign item | body temperature | 37.1±3.1 | 37.1±13.4 | 0.626 |
|  | pulse | 128.7±28.5 | 131.9±26.9 | <0.001 |
|  | Heart rate | 122.3±30 | 127.2±27.6 | <0.001 |
|  | Breathing rate | 35.2±29.1 | 36.0±71.7 | <0.001 |
|  | Blood oxygen saturation | 97.7±7.3 | 97.5±7.3 | <0.001 |
|  | Diastolic blood pressure | 56.2±16.4 | 54.5±15.4 | <0.001 |
|  | Systolic blood pressure | 97.5±19.3 | 96.7±18.8 | 0.001 |
| Lab test item | Subject activated partial thromboplastin time | 42.6±26 | 39.3±19.1 | <0.001 |
|  | Plasma D-dimer (DD) | 3.4±4.9 | 2.2±3.8 | <0.001 |
|  | Fibrinogen | 2.3±1.3 | 2.3±1.1 | 0.411 |
|  | International normalized ratio | 1.2±0.5 | 1.2±0.4 | 0.088 |
|  | Normal control activated partial thromboplastin time | 27.9±1.7 | 27.6±1.7 | <0.001 |
|  | Normal control prothrombin time | 11.8±0.3 | 11.8±0.5 | 0.022 |
|  | Normal control thrombin time | 19.1±0.6 | 19.1±0.6 | <0.001 |
|  | Prothrombin time (PT) | 13.8±5.8 | 14±5.2 | 0.07 |
|  | Thrombin time (TT) | 23.3±9.1 | 20.8±6.5 | <0.001 |
|  | Eosinophils | 0.9±1.7 | 1.1±2 | <0.001 |
|  | Neutrophil absolute value | 7.6±5.8 | 7.0±5.2 | <0.001 |
|  | Neutrophils (NE%) | 63.9±18.5 | 58.7±20.4 | <0.001 |
|  | Hematocrit | 32.4±5.9 | 33.1±6.2 | <0.001 |
|  | Hemoglobin | 104.7±19.4 | 107.5±20.8 | <0.001 |
|  | Absolute lymphocyte value | 2.5±1.8 | 3.1±2.2 | <0.001 |
|  | Lymphocyte (LY%) | 26.3±16.7 | 31.3±18.4 | <0.001 |
|  | Average hemoglobin | 28.3±2.5 | 28.4±2.6 | <0.001 |
|  | Mean hemoglobin concentration | 323.5±15.3 | 328.4±13.6 | <0.001 |
|  | Mean red blood cell volume | 87.4±7 | 86.6±7.1 | <0.001 |
|  | Mean platelet volume | 9.7±1.3 | 9.6±1.3 | <0.001 |
|  | Platelet packed volume | 0.3±0.1 | 0.3±0.1 | 0.951 |
|  | Platelet distribution width | 14.5±2.7 | 14.3±2.9 | <0.001 |
|  | Platelet count | 303.1±172.3 | 307.8±169.1 | 0.036 |
|  | Red blood cell count | 3.7±0.7 | 4.2±43.4 | 0.056 |
|  | Red blood cell distribution width | 15.5±2.9 | 14.7±2.5 | <0.001 |
|  | White blood cell count | 11.1±6.9 | 11.3±6 | 0.17 |
|  | Basophil absolute value | 0.0268±0.0329 | 0.0307±0.0354 | <0.001 |
|  | Absolute value of monocytes | 0.9±0.7 | 0.9±0.6 | 0.716 |
|  | Monocyte | 8.1±4.2 | 7.8±4.2 | <0.001 |
|  | Eosinophil absolute value | 0.1±0.2 | 0.2±0.3 | <0.001 |
|  | calcium | 1.2±0.1 | 1.2±0.2 | <0.001 |
|  | chlorine | 106.8±8.7 | 108.4±356.9 | 0.25 |
|  | Potassium | 3.6±0.7 | 3.7±0.7 | <0.001 |
|  | sodium | 138.2±8.2 | 137.4±6.2 | <0.001 |
|  | Bicarbonate | 27.4±5.3 | 25.4±4.7 | <0.001 |
|  | Methemoglobin | 0.9±0.3 | 0.9±0.3 | <0.001 |
|  | Partial pressure of carbon dioxide (PaCO_2_) | 43.7±12.9 | 41.2±10.6 | <0.001 |
|  | pH | 7.42±0.08 | 7.40±0.07 | <0.001 |
|  | Oxygen partial pressure (PaO_2_) | 131.8±58.1 | 145.3±68.5 | <0.001 |
|  | Standard base excess (SBE) | 3.1±5.6 | 1.0±5.1 | <0.001 |
|  | Oxygen saturation | 95.3±9.7 | 95.6±9.8 | <0.001 |
|  | Actual alkali surplus | 3.0±5.2 | 0.9±4.9 | <0.001 |
|  | Anion gap | 3.9±5.7 | 5.4±5.9 | <0.001 |
|  | Carboxyhemoglobin | 1.1±0.6 | 1.1±0.5 | <0.001 |
|  | Glucose (electrode method) | 7.1±2.9 | 9.3±3.3 | 0.221 |
|  | Lactic acid | 1.7±1.7 | 1.8±1.8 | <0.001 |
|  | Thrombin time (TT) difference | 4.1±9.1 | 1.7±6.5 | <0.001 |
|  | Prothrombin time (PT) difference | 2.0±5.8 | 2.2±5.2 | 0.05 |
|  | Time difference in activated partial thromboplastin | 14.7±25.9 | 11.7±19.3 | <0.001 |

**Table S2 The detail information of disease group**

| **Diseases group** | **Disease** |
| --- | --- |
| Bleeding | Subdural hemorrhage |
|  | Cerebral hemorrhage |
|  | Intracranial hemorrhage (unknown cause) |
|  | Traumatic epidural hemorrhage |
|  | Gastrointestinal hemorrhage |
|  | Non-traumatic subdural hemorrhage |
|  | Traumatic subarachnoid hemorrhage |
|  | Pulmonary hemorrhage |
|  | Hemorrhagic shock |
|  | Traumatic subdural hemorrhage |
|  | Brainstem hemorrhage |
| Cancer | Medullary junction malignant tumor |
|  | Mediastinal tumor |
|  | Spongiform hemangioma |
|  | Mediastinal malignant tumor |
|  | Adrenal tumor |
|  | Craniopharyngioma |
|  | Angiosarcoma |
|  | Endodermal sinus tumor |
|  | Intracranial tumors of undetermined nature |
|  | Retroperitoneal tumor |
|  | Renoblastoma |
|  | Angiogenic cell tumor |
|  | Tumor of undetermined temporal lobe dynamics |
|  | Neuroblastoma |
|  | Bronchial dynamic undetermined tumor |
|  | Choroid plexus papilloma |
|  | Malignant tumor of the stomach |
|  | Aneurysm |
|  | Ovarian tumor |
|  | Sarcoma |
|  | Cerebrovascular junction tumor |
|  | Ventricular meningioma |
|  | Brain Tumor |
|  | Hepatoblastoma |
|  | Burkitt's lymphoma |
|  | Liver Tumor |
|  | Lymphangioleioma |
|  | Teratoma |
|  | Parenchymal pseudopapillary tumor |
|  | Malignant tumor of the pterygoid saddle area |
|  | Metastatic endodermal sinus tumor |
|  | Tumor |
|  | Mediastinal dynamic unspecified tumor |
|  | Renal tumor |
|  | Coronary artery tumor |
|  | Renal malignant tumor |
|  | Benign tumor of the fourth ventricle |
|  | Temporal lobe tumor |
|  | Ovarian malignant tumor |
|  | Pancreaticoblastoma |
|  | Retroperitoneal malignant tumor |
|  | Benign tumor of tongue |
|  | Neurofibromatosis |
|  | Cystadenoma |
|  | Adrenal malignant tumor |
|  | Subventricular giant cell astrocytoma |
|  | Pelvic tumor |
|  | Thymus tumor |
|  | Transverse myxosarcoma |
|  | Rhabdomyosarcoma |
|  | Malignant lymphoma |
|  | Malignant nerve sheath tumor |
|  | Aortic sinus aneurysm |
|  | Thoracic tumor |
| CHD | Ventricular septal defect |
|  | Heart disease |
|  | Atrial septal defect |
|  | Congenital heart disease |
|  | Tetralogy of Fallot |
|  | Aortic arch stenosis |
|  | Arterial catheterization |
|  | Triatrial heart |
|  | Congenital atrial septal defect |
|  | Complete ectopic pulmonary vein drainage |
|  | complete transposition of the great arteries |
|  | Atrial septal defect |
|  | congenital transposition of the great vessels |
|  | congenital right-sided aorta |
|  | Congenital double aortic arch |
| Intracranial occupying lesions | Intracranial occupying lesions |
| Nonmalignant pathology | Cerebral cysts |
|  | Congenital cerebral cyst |
|  | Congenital Pulmonary Cyst |
|  | Common bile duct cyst |
|  | Epidural cyst |
|  | Pulmonary cyst |
|  | Retroperitoneal mass |
|  | Abdominal mass |
|  | [Brain] Arachnoid cyst |
|  | Saddle mass |
|  | Buccal mass |
|  | Pancreatic mass |
|  | Pelvic mass |
|  | Mediastinal cyst |
|  | Sacrococcygeal mass |
|  | Bronchial cyst |
|  | Brain abscess |
|  | Pulmonary mass |
|  | Brain swelling |
|  | Splenic cyst |
|  | Bile duct cyst |
|  | Congenital common bile duct cyst |
|  | Gluteal swelling |
|  | Pineal region swelling |
|  | Ovarian inclusion cyst |
|  | Cervical swelling |
|  | Rib swelling |
|  | Splenic swelling |
|  | Chest wall mass |
|  | Mesenteric cyst |
|  | Liver mass |
|  | Congenital third ventricular cyst |
|  | Benign tumor of cerebral bridge |
| Other congenital disease | Congenital spinal cord embolism syndrome |
|  | Congenital cystic lung |
|  | Funnel chest |
|  | Congenital megacolon like origin disease |
|  | megacolon |
|  | Post-operative megacolon enterostomy |
|  | Congenital anal anomalies |
|  | Personal history of congenital malformations, deformities and chromosomal abnormalities |
|  | Congenital atresia of the bile duct |
|  | Congenital ileal atresia |
|  | Congenital umbilical hernia |
|  | Congenital diaphragmatic hernia |
|  | congenital arteriovenous fistula of the trunk |
|  | Congenital spinal cord bulge |
|  | Congenital tricuspid valve subluxation malformation |
|  | Congenital tricuspid valve insufficiency |
|  | Congenital esophageal atresia |
|  | Congenital small bowel atresia |
|  | Congenital jejunal atresia |
|  | Congenital malformation of the upper gastrointestinal tract |
|  | Congenital chondrodysplasia of the larynx |
|  | Congenital laryngeal stridor |
|  | Congenital muscular defect of the stomach wall |
|  | Congenital anal agenesis, atresia and stenosis with fistula |
|  | Congenital hypertrophic pyloric stenosis |
|  | Congenital cranial agenesis |
|  | Congenital umbilical malformation |
| Premature | Premature infants |
|  | Retinopathy of prematurity |
| Infection or inflammation | Enteritis |
|  | Bronchopneumonia |
|  | Intrahepatic cholangitis |
|  | Pneumonia |
|  | Neonatal necrotizing small bowel colitis |
|  | Sepsis |
|  | Hepatitis |
|  | Neonatal pneumonia |
|  | Acute pancreatitis |
|  | Meningoencephalitis |
|  | Infective endocardial inflammatory bullae |
|  | Ulcerative colitis |
|  | Acute septic meningitis |
|  | Parasitic infection |
|  | Non-infectious multi-organ dysfunction syndrome (MODS) |
|  | Acute upper respiratory tract infection |
|  | Acute gangrenous appendicitis |
|  | Neonatal sepsis |
|  | Post-surgical pancreatitis |
|  | Biliary ductitis |
|  | Peritonitis |
|  | Staphylococcal pneumonia |
|  | Common bile duct stones with cholecystitis |
|  | Specific acute myocarditis |
|  | Constrictive pericarditis |
|  | Migratory pneumonia |
|  | Cholecystitis |
|  | Post-infectious cough |
|  | Cellulitis |
|  | Focal encephalitis |
|  | Common bile duct stones with chronic cholecystitis |
|  | Chronic fibrous pancreatitis |
|  | Infected bursitis of the thigh |
|  | Septic meningoencephalitis |
|  | Parapharyngeal space infection |
|  | Tonsil stump infection |
|  | Myocarditis |
|  | Viral encephalitis |
|  | Enterovirus infection |
|  | Disseminated encephalitis |
|  | Aspiration pneumonia |
|  | Neonatal aspiration pneumonia |
|  | Acute laryngitis |
|  | Acute pyogenic appendicitis |
|  | Urinary tract infection |
|  | Myositis |
|  | peri-splenitis |
|  | Endocarditis with aortic atresia insufficiency |
|  | Brainstem encephalitis |
|  | Adenovirus pneumonia |
|  | Choledocholithiasis with cholangitis |
|  | Pyogenic myelitis |
|  | EBV infection |
|  | Limited encephalitis |
| Other | Convulsions |
|  | Head trauma |
|  | Foreign body in the respiratory tract |
|  | Isolated lung |
|  | Hydrocephalus |
|  | Burn (scalding) injury |
|  | Inherited metabolic diseases |
|  | Pulmonary valve atresia |
|  | Adult respiratory distress syndrome |
|  | Malnutrition |
|  | Premature closure of cranial suture |
|  | 17α-hydroxylase deficiency |
|  | Subdural effusion |
|  | Fever to be investigated |
|  | Post-operative digestive system disorders |
|  | cerebrovascular specific malformation |
|  | Severe malnutrition |
|  | Pulmonary artery atresia |
|  | Skull defect repair |
|  | Bone fracture |
|  | Skull fracture |
|  | Smoker's disease |
|  | malrotation of the bowel |
|  | Abdominal pain pending investigation |
|  | Abnormal origin of coronary arteries |
|  | intestinal adhesions |
|  | Spina bifida |
|  | Encephalopathy |
|  | mediastinal hernia |
|  | Pulmonary valve stenosis |
|  | Adrenal cortical insufficiency |
|  | Complete atrioventricular septal defect |
|  | Pulmonary emphysema |
|  | Under examination and observation after traffic accident |
|  | Strangulated bowel necrosis |
|  | Arnold Chiari malformation (A-K syndrome) |
|  | Inguinal hernia |
|  | Perforation of the digestive tract |
|  | Jaundice |
|  | Disorders of electrolyte metabolism |
|  | Intestinal obstruction |
|  | Abdominal swelling |
|  | Pulmonary artery stenosis |
|  | Paroxysmal epilepsy |
|  | Esophageal atresia with tracheoesophageal fistula |
|  | Single atrium |
|  | Traumatic brain herniation |
|  | Respiratory distress |
|  | Drowning |
|  | Cardiomyopathy |
|  | Intussusception |
|  | Diaphragmatic hernia |
|  | Non-traumatic epidural hematoma |
|  | Asphyxia |
|  | Occipital foramen magnum hernia |
|  | Common bile duct dilatation |
|  | Respiratory failure |
|  | Myelopathy |
|  | Partial pulmonary vein ectopic drainage |
|  | Diaphragmatic elevation |
|  | Specific surgical follow-up medical treatment |
|  | Pericardial effusion |
|  | Aortic constriction |
|  | Esophageal compression |
|  | Neonatal vomiting |
|  | Vitamin K deficiency |
|  | Bile duct stenosis |
|  | Williams Syndrome |
|  | Extrusion syndrome |
|  | Spinal Cord Occupational Lesions |
|  | Diabetes mellitus |
|  | Splenic Injury |
|  | Renal Failure |
|  | Hepatobiliary duct dilatation |
|  | Pulmonary hypertension |
|  | Brain contusion |
|  | Mitochondrial encephalomyopathy |
|  | Interstitial lung disease |
|  | Liver Failure |
|  | Venous sclerosis |
|  | muffled fever syndrome |
|  | Motor vehicle collision with indeterminate intent |
|  | Cardiac arrhythmia |
|  | Successful resuscitation from cardiac arrest |
|  | Mesenteric dysplasia |
|  | Wavy diaphragm |
|  | Nausea and vomiting |
|  | Abnormal liver function |
|  | Influenza |
|  | Accidental poisoning by drug overdose |
|  | Occupational liver lesions |
|  | Routine well-child examination |
|  | Multiple burns with at least one third degree burn |
|  | Bilateral sensorineural deafness |
|  | Scalp laceration |
|  | Left coronary origin pulmonary artery |
|  | Skin contusion |
|  | Pulmonary artery sling |
|  | Chronic left heart insufficiency |
|  | Chicken chest |
|  | Fall, jumping or being pushed from a height with uncertain intent |
|  | Fall or fall from a house or building structure, fall from a building |
|  | Pneumothorax |
|  | Central hypoventilation |
|  | Persistent epilepsy |
|  | Heart failure after cardiac surgery |
|  | Pulmonary laceration |
|  | Bone marrow suppression after chemotherapy |
|  | Neonatal asphyxia |
|  | Bee stings (poisonous insect bites) |
|  | Common atrium |
|  | Airway obstruction due to inhalation or swallowing of sputum or foreign bodies |
|  | Mitral and aortic valve disorders |
|  | Injuries to persons in vehicle accidents |
|  | Neonatal hyperbilirubinemic encephalopathy |
|  | Gallbladder stones |
|  | Myoepithelial carcinoma |
|  | frontal bone fractures |
|  | Atrioventricular anomalous channel |
|  | Ventriculoperitoneal shunt dislocation |
|  | Incarcerated inguinal hernia |
|  | Heart Failure |
|  | Aortic stenosis |
|  | Intestinal rupture |
|  | Circumferential pancreas |
|  | Aortic stenosis |
|  | Hepatic insufficiency |
|  | Protein-losing enteropathy |
|  | Vomiting of blood |
|  | Langerhans cell histiocytosis |
|  | Brain bulge |
|  | Poisoning by ingestion of poisonous mushrooms |
|  | Coronary artery occlusion |
|  | Scalp hematoma |
|  | Hemophagocytic syndrome |
|  | Intestinal duplication malformation |
|  | Anaphylaxis |
|  | Subcutaneous nodules |
|  | Pulmonary abscess |
|  | Cerebrovascular disease |
|  | Abnormal pulmonary venous connection |
|  | Splenomegaly |
|  | Tricuspid valve disease |
|  | Neonatal ABO hemolysis |
|  | Massive alveolar emphysema |
|  | Laryngeal obstruction |
|  | Hidradenitis |
|  | cardia flaccid inability |
|  | Paraplegia |
|  | Cranial separation |
|  | periodic paralysis |
|  | Accidental drug poisoning and exposure to such drugs |
|  | Traumatic lower limb amputation |
|  | Pneumothorax |
|  | Short bowel syndrome (post-intestinal resection syndrome) |
|  | Hypertension grade 1 |
|  | Hyponatremia |
|  | Intestinal torsion |
|  | Ureteral diverticulum |
|  | Double outlet of right ventricle |
|  | Shock |
|  | Alcohol intoxication |
|  | Coma |
|  | Specific tricuspid valve disease |
|  | Neonatal bronchopulmonary dysplasia |
|  | Hereditary spherocytosis |
|  | Neonatal pathologic jaundice |
|  | Microcephaly |
|  | Adnexal torsion |
|  | Triadic rhythm [ventricular prematureness] |
|  | Intestinal perforation (non-traumatic) |
|  | Acute hemolytic anemia |
|  | Acute Renal Failure |
|  | Anemia |
|  | Cyanosis |
|  | Cerebral anoxia |
|  | Neonatal respiratory distress syndrome |
|  | Common bile duct obstruction |
|  | Increased intracranial pressure |
|  | Fecal occult blood |
|  | Torsion of the greater omentum with necrosis |
|  | Skin rash |
|  | Incarcerated inguinal hernia with obstruction |
|  | Specific pesticide toxic effects (crops) |
|  | Skin abscess |
|  | Neonatal hypoglycemia |
|  | Granuloma of the small intestine |
|  | Post-operative hypocardial discharge after precordial surgery |
|  | Gastrointestinal foreign body |
|  | Headache to be investigated |
|  | Lung shadow |
|  | hyperammonemia |
|  | Peritoneal effusion |
|  | Actinic nerve palsy |
|  | Carcinomatous cerebral leukomalacia |
|  | Neonatal intestinal obstruction |
|  | Carbon monoxide toxic effects |
|  | Cirrhosis of the liver |
|  | Hepatogenic heart disease |
|  | Otawara syndrome |
|  | Neonatal abdominal distention |
|  | Removal of internal fracture fixation device |
|  | Congestive heart failure |
|  | Abnormal renal function |
|  | Cerebrovascular arteriovenous malformation |
|  | Gallbladder polyps |
|  | Behçet's syndrome |
|  | Intentional self-poisoning and exposure to chemical agents and harmful substances |
|  | Portal hypertension |
|  | portal hypertension |
|  | Acute febrile mucocutaneous lymph node syndrome (Kawasaki disease) |
|  | Feeding disorders in infancy and childhood |
|  | Specific generalized epilepsy and epilepsy syndrome |
|  | Parapharyngeal abscess |
|  | Acute liver failure |
|  | Crohn's disease |
|  | Bile duct atresia |
|  | Uroplasia |
|  | Sciatic fracture |
|  | Intentional self-poisoning by drugs and exposure to such drugs |
|  | Acute respiratory failure |
|  | Methylmalonic acidemia |
|  | Hypertrophic non-obstructive cardiomyopathy |
|  | Septic shock (not associated with organ failure) |
|  | Hand, foot, and mouth disease (suspected) |
|  | Toxic effects of pesticides |
|  | Constipation |
|  | Tracheal stenosis |
|  | Acquired brain malformation |
|  | Pulmonary insufflation (atelectasis) |
|  | Skull base fracture |
